# Advances in PET/CT Imaging for Breast Cancer Patients and Beyond

**DOI:** 10.3390/jcm12020651

**Published:** 2023-01-13

**Authors:** David Khalil, Andrew Lotfalla, Antoine Girard, Richard Ha, Laurent Dercle, Romain-David Seban

**Affiliations:** 1 Campbell University School of Osteopathic Medicine, Lillington, NC 27546, USA; 2 Touro College of Osteopathic Medicine, Middletown, NY 10940, USA; 3 Department of Nuclear Medicine, CHU Amiens-Picardie, 80000 Amiens, France; 4 Department of Radiology, NewYork-Presbyterian, Columbia University Irving Medical Center, New York, NY 10032, USA; 5 Department of Nuclear Medicine, Institut Curie, 92210 Saint-Cloud, France

## 1. Introduction

Breast cancer is the most common cancer in women around the world and the fifth leading cause of cancer-related death [1]. A current challenge is performing early and personalized management based on diagnostic tools and associated biomarkers. Among these, [18F]Fluorodeoxyglucose Positron Emission Tomography with Computed Tomography ([18F]FDG PET/CT) imaging currently plays a crucial role in determining the extent of the disease [2,3] and in ensuring that the treatment is effective [4]. However, the arrival of new treatments, the discovery of new therapeutically actionable pathways, and the advent of new specific radiotracers in nuclear medicine is opening up many opportunities for precision medicine approaches guided by PET/CT imaging.

## 2. Predictive/Prognostic [18F]FDG PET Biomarkers for Novel Therapies

New therapeutic molecules, as well as antibody–drug conjugates (trastuzumab–deruxtecan and sacituzumab govitecan for example) [5,6] or molecule combinations (chemo-immunotherapy for example) [7,8,9], have revolutionized the management of metastatic breast cancer patients, whose prognosis is among the poorest. Given that there is no identified effective predictive or prognostic biomarker yet for such novel therapies, it is of paramount importance to determine whether [18F]FDG PET/CT imaging could contribute to the optimization of patient management.

### 2.1. Antibody–Drug Conjugates

Antibody–drug conjugates have been shown to be an effective treatment for breast cancer. The role of [18F]FDG PET/CT imaging for the assessment of response to treatment remains an area of active investigation.

### 2.2. Immune Checkpoint Inhibitors (Plus Chemotherapy)

In the Impassion 130 trial, metastatic triple-negative breast cancers that were Programmed Death-Ligand 1 (PD-L1)-positive and were treated with atezolizumab and nab-paclitaxel had a longer overall survival with treatment. [18F]FDG PET/CT is a promising theranostic tool for first-line immunotherapy [10] in patients with metastatic triple-negative breast cancer because increased tumor metabolism is associated with poorer prognosis and response to treatment. Additionally, several studies have demonstrated an association between the glucose metabolism within the tumor and hematopoietic tissues and the systemic immunosuppressive environment for predicting dismal response to immunotherapy.

### 2.3. CDK 4/6 Inhibitors (Plus Endocrine Therapy)

Cyclin-dependent 4/6 kinase inhibitors plus endocrine therapy is considered the gold standard treatment for individuals with hormone receptor-positive Human epidermal growth factor receptor 2 (HER2-positive) breast cancer [11]. According to previous studies, low levels of whole-body metabolic tumor volume (MTV), total lesion glycolysis (TLG), and maximum standardized uptake value (SUVmax) on [18F]FDG PET/CT are significantly associated with prolonged progression-free survival and overall survival in patients treated with endocrine therapy alone or in combination with Cyclin-dependent Kinase 4/6 (CDK 4/6) inhibitors [12].

## 3. Deciphering Pro-Tumoral Phenotypes Using [18F]FDG PET/CT Imaging

Recent works suggest that measuring metabolic activity in non-tumor tissues could provide valuable biomarkers, such as measuring metabolism in lymphoid tissues (bone marrow and spleen) that are in close communication with the tumor microenvironment, to predict a patient’s response to certain therapies [13,14,15]. Although there are some avenues for elucidating the underlying pathophysiological mechanism [16,17,18], the joint study of mouse models with breast cancer using [18F]FDG PET/CT using a “micro-PET” device dedicated to rodent studies would provide a better basis for deciphering pro-tumoral phenotypes and for better understanding therapeutic resistance in humans.

## 4. Innovative PET Tracers for Personalized/Precision Medicine

The advent of innovative PET radiotracers such as FluoroEStradiol labeled with 18-Fluor ([18F]FES) [19,20,21] or Fibroblast-Associated Protein Inhibitor labeled with 68-Gallium ([68Ga]FAPI) [22,23,24] is expected to improve the personalization of patient management, optimize therapeutic choices, and improve outcomes in the field of breast cancer.

### 4.1. Limitations of [18F]FDG PET

[18F]FDG is not a cancer-specific tracer. Benign diseases that are related to infection or inflammation can show false-positive [18F]FDG uptake causing unnecessary or excess treatment [25,26].

### 4.2. FluoroEStradiol Labeled with 18-Fluor ([18F]FES) PET/CT Imaging

[18F]FES has been shown to be an effective molecular imaging technique in the detection of in vivo estrogen receptor (ER) expression due to its high specificity and sensitivity for ER-positive lesions and can be used as a predictor of patient response to hormonal therapy [27], making the advent of [18F]-FES pivotal in making these distinctions to guide treatment [27]. In order to optimize personalized treatment, the ER expression and an estimate of the heterogeneity in the ER expression in metastatic lesions must be determined [20,28]. However, this cannot accurately be performed using metastasis biopsy or standard imaging with [18F]FDG. [18F]FES PET has been shown to determine ER heterogeneity and guide personalized treatment options [20]. The U.S. Food and Drug Administration (FDA) has recently approved [18F]FES as a PET tracer for the evaluation of ER heterogeneity, indicating the potential impact [18F]FES PET has on major healthcare decisions [29].

Invasive lobular carcinoma is considered to be the fifth most common cancer overall in females and the second most common subtype of breast cancer malignancy, only behind invasive breast carcinoma of no special type (NST) [29]. In invasive lobular carcinoma, bone metastases are common and can be more easily and readily visualized in [18F]FES. [18F]-FES PET was found to be much more sensitive to the detection of osseous lesions than [18F]FDG PET [29]. [18F]FES PET is integral in helping diagnose the progression of ILC and can help navigate treatment modalities as well as treatment efficacy [29]. [18F]FES is extracted and metabolized by the liver, which complicates its applicability for breast cancer staging in liver metastases because of the high [18F]FES uptake [27,30]. In this case, different imaging modalities such as CT or MRI with contrast would be preferred [29].

### 4.3. Fibroblast-Associated Protein Inhibitor Labeled with 68-Gallium ([68Ga]FAPI) PET/CT Imaging

Fibroblast activation protein (FAP) is a serine protease from the dipeptidyl peptidase-4 (DPP-4), which functions in tumor stroma and is highly specific and sensitive for the detection of many carcinomas [31]. FAP is expressed in various tumors and contributes to migration and tumor angiogenesis [32].

[68Ga]FAPI PET/CT is a new radiotracer targeting FAP in order to visualize tumor stroma and detect metastases in various organ systems of the body [33]. [68Ga]FAPI PET/CT is considered to be superior to [18F]FDG in detecting more lesions and showing tumor activity. [68Ga]FAPI has higher SUVs than [18F]FDG in lesions located in primary breast, local/distant lymph node metastasis, liver metastasis, lung metastasis, and bone metastasis. [68Ga]FAPI was able to detect lesions within one month of the post-chemotherapy period, which is not the case for [18F]FDG [31]. In comparison to [18F]FDG PET/CT, [68Ga]FAPI was able to more accurately depict primary and metastatic lesions, specifically in the liver, abdomen, and brain, because of its high target-to-background ratios (TBRs) and good tumor delineation. Detecting distant metastasis through detecting more lesions can lead to changes in treatment approach [31].

In tumors smaller than 1 cm, [68Ga]FAPI PET/CT demonstrates a higher uptake and therefore has the ability to reveal more lesions in comparison with [18F]FDG PET/CT [34]. This allows for a greater overall detection of lesions. [68Ga]FAPI PET demonstrates a superior uptake in mediastinal and abdominal lymph nodes [33]. The SUV values of lymph nodes on [68Ga]FAPI PET/CT were higher than [18F]-FDG PET/CT when comparing lymph node uptake. Histopathological confirmation of lymph nodes reveals that [68Ga]FAPI has a higher sensitivity and accuracy but a lower specificity when compared to [18F]FDG in detecting axillary lymph nodes. This is because FDG fails to demonstrate uptake in micrometastases [33]. A major limiting factor to [68Ga]FAPI is its relative inaccessibility when compared to [18F]-FDG. [18F]FDG has been widely used in the detection of cancer in multiple body systems and is therefore posed as a cost-effective imaging alternative in comparison to [68Ga]FAPI PET/CT [35].

### 4.4. HER2 PET Imaging

Between 15% and 20% of breast tumors are Human epidermal growth factor 2 (HER2)-positive cancers (with amplification or overexpression). These cancers are more aggressive than HER2-negative breast cancers and are treated with specific drugs that target HER2. Therefore, accurate characterization of HER2 expression can prevent overtreatment [36]. PET targeting HER2 may help visualize the heterogeneity of HER2 expression and allow a whole-body assessment of lesions that are not captured by single-site biopsy or standard imaging.

To date, most published studies mainly focused on HER2-positive breast cancer. HER2 PET using [89Zr]Trastuzumab has been used to help guide clinical decision making [37]. Other studies suggest a role for [64Cu]DOTA-trastuzumab PET/CT in optimizing and identifying patients with metastatic breast cancer who will benefit from HER2-targeted therapies [36,38]. The main disadvantage of these radiolabeled antibodies is that images need to be performed a few days after tracer injection. Specific radiotracers, including HER2-targeting affibody molecules [39] or nanobodies [40], are currently under development to allow imaging within hours of tracer administration and reduce radiation dose to patients.

## 5. Conclusions

While [18F]FDG PET is the current cornerstone of molecular imaging to personalize the care of patients with a diagnosis of breast cancer, PET with FluoroEStradiol labeled with 18-Fluor ([18F]FES), Fibroblast-Associated Protein Inhibitor labeled with 68-Gallium ([68Ga]FAPI), or targeting Human epidermal growth factor 2 (HER2)-positive tumors will be prospectively evaluated in multicenter clinical trials. The hypothesis is that these three novel radiotracers could assist oncologists in giving the right patient the right treatment at the right time, consequently avoiding the administration of unnecessary or potentially toxic treatments. In particular, the goal is to identify patients that would benefit from immunotherapies (using [68Ga]FAPI PET), chemotherapy versus endocrine therapy (using [18F]FES PET), or HER2-targeted therapies (using PET targeting HER2).
